# Can golfers choose low-risk routes in steep putting based on visual feedback of ball trajectory?

**DOI:** 10.3389/fspor.2023.1131390

**Published:** 2023-08-22

**Authors:** Yumiko Hasegawa, Ayako Okada, Keisuke Fujii

**Affiliations:** ^1^ Faculty of Humanities and Social Sciences, Iwate University, Iwate, Japan; ^2^ Japan Ladies Professional Golfers’ Association, Tokyo, Japan; ^3^ Graduate School of Informatics, Nagoya University, Aichi, Japan; ^4^ RIKEN Center for Advanced Intelligence Project, RIKEN, Fukuoka, Japan; ^5^ PRESTO, Japan Science and Technology Agency, Kawaguchi, Japan

**Keywords:** task redundancy, decision-making, risk, kinematics, ball trajectory, tour professional, intermediate golfer

## Abstract

This study aims to clarify why the aiming method in golf putting in risky situations differs based on skill level. This study set up a difficult challenge (steep slopes and fast ball rolling greens), which required even professional golfers to change their aim. A total of 12 tour professionals and 12 intermediate amateurs were asked to perform a steep-slope task with no visual feedback of outcomes (no FB) followed by a task with visual feedback (with FB). The aim of the task was for the ball to enter the hole in one shot. Additionally, the participants were told that if the ball did not enter the hole, it was to at least stop as close to it as possible. The participant's aim (as an angle) and the kinematics of the putter head and ball were measured. The results indicated that professionals' highest ball trajectory points were significantly higher than that of amateurs, especially with FB. Additionally, professionals had higher ball-launch angles (the direction of the ball when the line connecting the ball and the center of the hole is 0 degrees) and lower peak putter head velocities than amateurs. Furthermore, the aim angle, indicating the golfer's decision-making, was higher for professionals under both conditions. However, even with FB, the amateurs' aim angles were lower and the difference between trials was smaller than that of professionals. Therefore, this study confirmed that the professionals made more drastic changes to their aim to find low-risk routes than the amateurs and that the amateurs’ ability to adjust their aim was lower than that of professionals. The results suggest that the reason for the amateurs' inability to find low-risk routes lies in their decision-making. The professionals found better routes; however, there were individual differences in their routes.

## 1. Introduction

When throwing, kicking, or hitting a ball, the method of aiming significantly determines the outcome. Even when there are multiple solutions to achieve an objective, the risks involved should be considered when aiming. However, previous studies reported that even when learning improved performers' motor skills, it did not necessarily lead to an effective action plan (1). This study used the steep golf putting task to examine the aiming problem (goal setting), which is important in the context of analyzing performance under risk.

Route and aim planning are important skills in long-duration sports, such as tennis, golf, and free climbing. These sports require performers to plan their own aiming. In free climbing, performers imagine beforehand how to carry out the route and climbing movements (2), and performers who visualize more routes during observations have higher fluency (3). Previous studies found that experts paid more attention to the functionality of the climbing wall and holds during observation time than non-experts (4), and they disregarded the route that was considered unclimbable, compared to easy or difficult routes that were considered climbable (2). These findings indicated that experts pay more attention to the environment's physical characteristics, calculate environmental risks, and can reflect on motor planning and actual movement more than non-experts.

“Green reading” is necessary for golf putting, which has similar characteristics to route observation in climbing. Different skill level players have different green reading (5) and environmental perception abilities (6). Compared to professionals, when amateurs putt on steep slopes on golf courses, putts more often deviate from the front lower side toward the hole. Golf putting on steep slopes is more difficult than on relatively flat surfaces. Dias et al. (7) suggested that the motor control strategy for various golf-putting distances in the no-incline condition showed a linear adjustment of the kinematic variables, whereas the incline condition did not show such a linear adjustment. The results suggest that steep putting is more complex owing to the increased choice of launch angle and velocity combinations. Thus, there is considerable redundancy in the combination of launch angle and club head velocity on steep slopes. If a slope is steep, stopping the ball after hitting it is difficult. Consequently, in steep putting, if a golfer fails to hole-in on the first ball, there is a high risk that the next putt will also not hole-in. For example, if a performer chooses the wrong launch angle and speed, that is, if the angle is smaller and the speed is higher, the distance of the next putt would be longer if the ball is not a hole-in-one. Therefore, the aiming method is important for avoiding risks. However, only a few studies have focused on these problems. Golfers must consider the risk of missing the first putt and how much force to use to hit the ball in a specific direction.

Hasegawa et al. (6) examined golfers' perception of slopes and found that intermediate golfers had a lower perceptual resolution than professional golfers. That is, most professional golfers can accurately perceive the putting surface, while intermediate golfers underestimate them. Consequently, their aiming is significantly smaller than that of professionals. Such lower perceptual resolution influences decision-making and leads to incorrect aiming (6). However, it is unclear whether the problem lies in the golfers' decision-making. To investigate this issue, this study set two conditions: with no visual feedback (no FB) and with visual feedback (with FB) of the trajectory of the ball. Measuring participants' aim direction in no FB includes both their perceptual and decision-making abilities. That is, by setting the no FB condition, the golfer's ability to perceive the environment and motor execution based on their previous experiences can be observed. By setting the with FB condition, golfers can see the trajectory of the rolling ball, and based on that feedback, they may change their route to a less risky one.

With FB, golfers have the chance to correct their aiming point because they can continue attempting while examining the outcomes. Therefore, even if amateurs misperceive the slope, they may have the same aim as professionals. If amateurs do not change their aim to the same direction (angle) as that of professionals, even with FB, then it could be understood that there may be problems, not only in their ability to perceive the environment but also in their decision-making. Measuring the professionals' aims and performances may allow for a better understanding of the process that professionals employ to change their aim to a less risky route using visual feedback. Therefore, this study set up a difficult challenge (steep slopes and fast ball rolling greens), in which even professional golfers were required to change their aim. This study assumed that both professionals and amateurs would modify their aim under conditions of FB compared to no FB. If amateurs can increase their aim to the same level as professionals, it can be concluded that their problem lies primarily in their weaker ability to perceive the environment. Alternatively, if amateurs cannot increase their aim to the same level as professionals, it can be concluded that their decision-making is at fault. This study did not measure environmental perceptual ability as reported in previous studies (6). This study aimed to identify how golfers change their aim based on feedback as they examine the ball's trajectory.

Furthermore, this study aimed to clarify why aiming in golf putting in risky situations differs based on skill level. In other words, this study examined decision-making under risk, which has not been adequately considered in sports (1). Professional and amateur golfers were recruited and their putter heads and ball kinematics were measured. A golf-putting task with steep inclines and fastball rolls that experimentally increased the risk was set up and the aim, launch angle, and putter head speed, based on previous studies [e.g., (8)]; (6, 9–12), were examined. Additionally, the kinematics of the ball trajectory were examined. Performers use internal and external feedback to adjust their next performance based on the experiences of previous performances (13). The ball trajectory is considered an important external feedback factor; therefore, this study captured the trajectory of the ball.

## 2. Method

### 2.1. Participants

The sample consisted of 12 tour professionals playing in the Japan Golf Tour or Ladies Professional Golf Association golf tournaments and 12 amateur golfers whose average age was 35.8 ± 6.1 and 47.5 ± 7.4 years, with golfing experience of 24.2 ± 6.9 and 19.3 ± 9.1 years, respectively. The amateurs were intermediate players with an average score of 88.3 ± 2.5 for 18 holes. All participants were right-handed with normal or corrected-to-normal vision. All participants provided written informed consent after receiving an explanation of the study. All experimental procedures were approved by the Ethics Committee of Iwate University and conformed to the principles of the Declaration of Helsinki.

### 2.2. Task and apparatus

The task included a 3.0 m straight line distance between the ball and hole under 3-degree conditions with no visual feedback (no FB) and with visual feedback of outcomes (with FB). The 3-degree condition refers to the steepest slope among the greens of general golf courses and was also utilized in a previous study (14). For both conditions, a slice line tilted to the right relative to the hitting direction was set based on the empirical finding that most golfers are not proficient in slice lines, compared to hook lines (tilted to the left) (6). The participants' goal was to get a ball into a hole of the same size as that on an actual golf green (10.8 cm). As a task constraint, the participants were asked to get the ball as close to the target as possible, even if they did not hit a hole-in-one, similar to when playing on a golf course. Additionally, the participants were asked to indicate the aim point from which the ball would be launched before putting. Participants did not receive any explicit slope or distance information.

An artificial turf was designed for golf putting (Superbent; Newtons Inc., Kochi, Japan). The stimp rating, indicating the speed of the putting green, was approximately 12 ft. The ball-to-hole line was approximately 19.3 degrees to the line parallel to the side of the platform and to the right of the ball in the hitting direction. Owing to the limited size of the putting platform (5.0 m long × 3.6 m wide), it was not possible to capture the final ball position. Participants could move around freely in the green reading area, although the putting platform was a restricted area (see Supplementary Figure S1).

All participants wore instant shielding goggles in the no FB condition (AO-FOS; Applied Office Co., Ltd., Tokyo, Japan), limiting their field of view to 0.4 m in front of the ball. The system was shielded when the ball crossed the light between the photoelectric sensors. The goggle lens was transparent when there was no shutter, and the field of view was clear from up to six feet away. The participants' aim points, putter head, and ball kinematics were recorded using nine optical motion-capture cameras (OptiTrack Prime13; Acuity Inc., Tokyo, Japan) operating at 240 Hz. Twelve-millimeter reflective markers were attached to the toe, heel, and neck of the putter head. Additionally, a reflective sheet was attached to the ball to capture its trajectory (6). The root mean square error of the static and dynamic calibrations was <0.1 cm for all sessions. All participants used the same putter (SB-01HB; PRGR Corp., Yokohama, Japan) and balls (Srixon Z-Star XV; Dunlop Sports Co., Ltd., Hyogo, Japan).

### 2.3. Procedure

Participants first attempted the no FB condition, followed by the FB condition. Participants practiced with ten balls in the waiting room to familiarize themselves with the artificial turf under each condition. During the familiarization sessions, the surface was flat, with no slope. Participants were asked to wear shielding goggles during the session; however, their field of view was unobstructed after hitting. The participants then moved to the experimental area and received the following instructions: “Please try to hit ten balls “here”. The goal is to get the ball into the hole. However, if it does not enter the hole, please try to get the ball as close to the hole as possible. This is the distance you practiced earlier. Feel free to move around for the next three minutes and read the putting line.” Regarding the teaching “to get the ball as close to the hole as possible,” this study called it “task constraint”. Three minutes later, the participants put on the instant shielding goggles in the no FB condition. Following this, they were instructed on how to set the ball. Afterward, they crouched behind the ball toward the target, as they would in actual play, and indicated their intended ball launch direction. The researcher moved the aim point marker while following the participants' instructions and adjusted it until they believed it was in the perfect position. The researcher captured the marker position and attached a circular white sticker (0.8 cm diameter) to the artificial turf at the same position as the aim point marker. Because the artificial turf was uniformly green, it allowed the participants to maintain sight of the direction. Subsequently, the participants practiced once to familiarize themselves with their inhibited field of view immediately after the hit. After the participants hit the ball, the white stickers were removed. In the no FB condition, immediately after the participant hit the ball, the field of view was obscured by the system. After the experimenter retrieved the ball and was ready for the next trial, the occluded goggles returned to their original, transparent state. The participants repeated this process ten times. After the no FB condition, participants took a 15-minute break before attempting the FB condition.

### 2.4. Dependent variables

All digitized data were smoothed with a fourth-order Butterworth filter (5-Hz cut-off) based on the root mean square of the residual error between the original and smoothed data (15, 16). The highest point of the ball trajectory in the anteroposterior direction (APD) and the mediolateral direction (MLD) and the ball speed near the hole (when crossing the coordinate line at the left end of the hole) were obtained. In addition, the peak speed of the putter head during the downswing and ball launch angles that affected the ball trajectory were obtained (6). Previous studies indicated peak velocity occurs just before impact [e.g., (17)] because the putter head velocity drops when the putter head hits the ball. Peak velocity is also highly correlated with the distance the ball rolls (18). The ball launch angle was determined using the direction of the launched ball with a line connecting the ball and the center of the hole at 0 degrees (ball-hole line), that is, the average angle for 0.1 s after ball collision. The aim point was calculated using the angle based on the ball-hole line. Furthermore, to examine the changes between trials, the difference values of the aim angles with FB were calculated. Further, Table 1 presents the dependent variables of this study.

**Table 1:** List of dependent variables in this study.

No.	Variable name	Note
1	The highest point of ball trajectory (m)	In terms of anteroposterior direction (APD) and mediolateral direction (MLD)
2	Ball speed near the hole (m/s)	The value when crossing the coordinate line at the left end of the hole
3	Aim angle (°)	The angle of the aim when the line connecting the ball and the center of the hole (ball-hole line) is 0°
4	The difference values between trials of the aim angle (°)	The value of the next trial minus the value of the previous trial, only for the FB condition
5	Ball launch angle (°)	Ball launch angle from the ball-hole line
6	Peak speed of putter head (m/s)	Combining the velocities along the x- and y-axes

### 2.5. Statistics

A two-factor mixed-design analysis of variance (ANOVA) was performed to explain the relationship between the two groups (professional and amateur) and the two conditions (no FB and FB) for the highest point for APD and MLD, ball speed near the hole, peak speed of the putter head, ball-launch angle, and aim angle. Regarding the difference values between trials concerning aim angle with FB, a two-factor ANOVA was conducted to explain the relationship between the two groups and the nine variations (2-1, 3-2, 4-3, 5-4, 6-5, 7-6, 8-7, 9-8, 10-9). The difference values were repeated-measures factors, and multiple comparison tests were performed using Bonferroni's method. Welch's t-test was performed for each condition to verify the differences between the groups regarding the ball-launch angle and peak velocity of the hole-in trials. The “*f*” and “*d*” values were calculated as effect-size indices for the ANOVAs (19) and Welch's *t*-test. According to Cohen's (20) conventions, small (*f* = 0.10, *d* = 0.20), medium (*f* = 0.25, *d* = 0.50), and large (*f* = 0.40, *d* = 0.80) effect sizes were reported. All data were analyzed using PASW Statistics (ver. 18.0; IBM Japan Ltd., Tokyo, Japan). The alpha level of significance was set at *p* < .05; however, statistical results with effect sizes greater than medium were also mentioned. Additionally, a power analysis was conducted (see Supplementary text) and confirmed that all 1-β were between 0.8–1.0.

## 3. Results

### 3.1. Ball trajectory and ball speed near the hole

**Figure 1:**
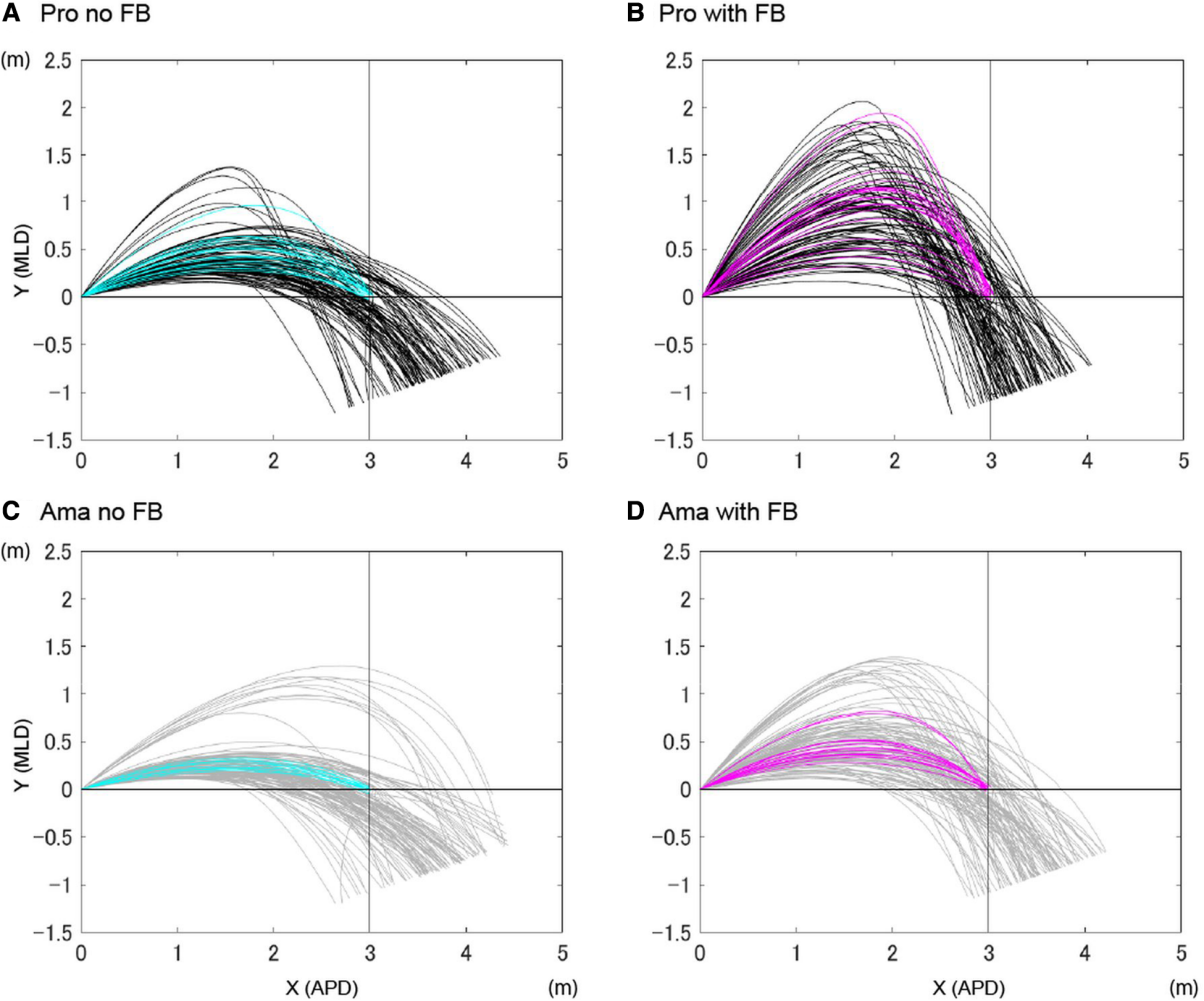
Participants’ ball trajectories. (**A**) shows the ball trajectories of professionals in the no FB condition. (**B**) shows the ball trajectories of professionals in the FB condition. (**C**) shows the ball trajectories of amateurs in the no FB condition. (**D**) shows the ball trajectories of amateurs in the FB condition. Cyan and magenta indicate hole-in trials. APD indicates the anteroposterior direction. MLD indicates the mediolateral direction.

**Figure 2:**
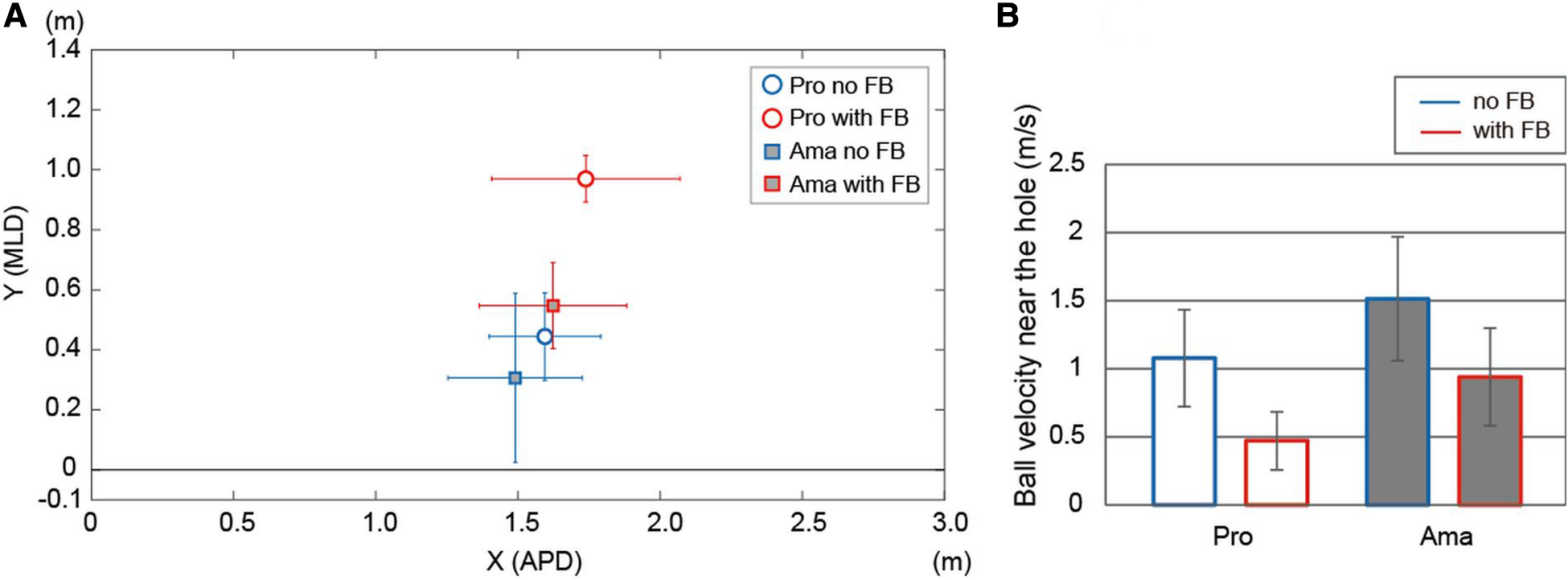
The average highest points and the ball velocities near the hole. (**A**) indicates the average value of the highest point of the ball trajectories under both conditions in both groups. Error bars indicate ±1 SD. (**B**) shows the ball velocity near the hole results. APD indicates the anteroposterior direction. MLD indicates the mediolateral direction.

Figures 1A–D presents all ball trajectories. The ANOVA results for the highest point for APD (Figure 2A) indicated that the interaction was not significant. However, the main effect of the condition (*F*_1,22_ = 53.00, *p* = 0.002, *f* = 0.74) was significant; the highest point for the APD in the FB condition was larger than that in the no FB condition, regardless of skill level. The main effect of the group was not significant.

The ANOVA results for the highest point for MLD (Figure 2A) indicated that the interaction was significant (*F*_1,22_ = 5.67, *p* = 0.03, *f* = 0.51). Simple-effects testing indicated that the highest point for MLD of professionals in the FB condition was higher than that of amateurs (*F*_1,22_ = 11.09, *p* = 0.003, *f* = 0.71). The highest point for MLD of professionals and amateurs was higher with FB than with no FB (pro: *F*_1,22_ = 38.66, *p* = 2.91 × 10^−6^, *f* = 1.33, ama: *F*_1,22_ = 8.13, *p* = 0.009, *f* = 0.61).

The ANOVA results for ball speed near the hole (Figure 2B) indicated that the interaction was not significant. However, the main effects of group (*F*_1,22_ = 12.97, *p* = 0.002, *f* = 0.76) and condition (*F*_1,22_ = 62.81, *p* = 6.90 × 10^−8^, *f* = 1.68) were significant. The professionals' ball speed was lower than that of amateurs, and the ball speed in the FB condition was lower than that in the no FB condition, regardless of skill level.

### 3.2. Aim angle and the difference values between trials

**Figure 3:**
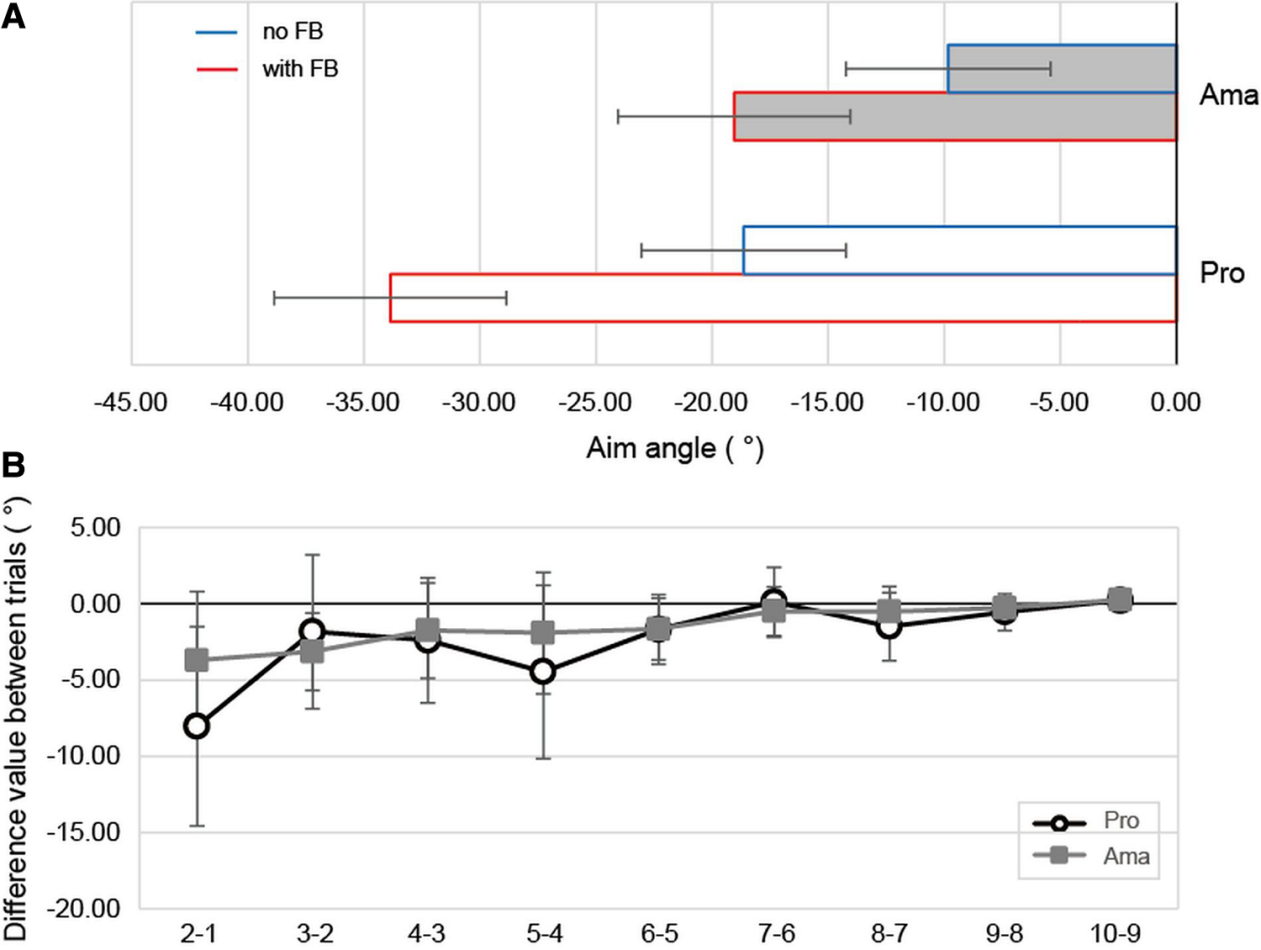
The results of the aim angle and the difference values between aim angle trials. (**A**) shows the average aim angle of each group. (**B**) shows the difference values between aim angle trials. Negative values indicate the left side of the ball-hole line and positive values indicate the right side.

The results of the ANOVA regarding the aim angle (Figure 3A) indicated that the interaction was significant (*F*_1,22_ = 3.25, *p* = 0.09, *f* = 0.38). Simple-effects testing indicated that the aim angle in the FB condition was larger than that in the no FB condition in both groups (pro: *F*_1,22_ = 41.87, *p* = 1.64 × 10^−6^, *f* = 1.38, ama: *F*_1,22_ = 15.38, *p* = 7.30 × 10^−4^, *f* = 0.84). Additionally, both the no FB (*F*_1,22_ = 7.14, *p* = 0.01, *f* = 0.57) and FB conditions (*F*_1,22_ = 15.41, *p* = 7.23 × 10^−4^, *f* = 0.84) were significant in both groups; the aim angles of professionals and amateurs were significantly different, and the aim angles of professionals were larger than those of amateurs. The main effects are described in the supplementary text.

The ANOVA results for the difference values between trials regarding aim angle (Figure 3B) indicated that the interaction was not significant. However, the main effect of the group was significant (*F*_1,22_ = 3.02, *p* = 0.09, *f* = 0.37); the difference in values for professionals was larger than that for amateurs. Additionally, the main effect of the variation was significant (*F*_1,22_ = 7.74, *p* = 0.01, *f* = 0.59). The results of multiple comparisons are presented in Supplementary Table S1.

### 3.3. Ball launch angle, peak speed of putter head, and hole-in trials

**Figure 4:**
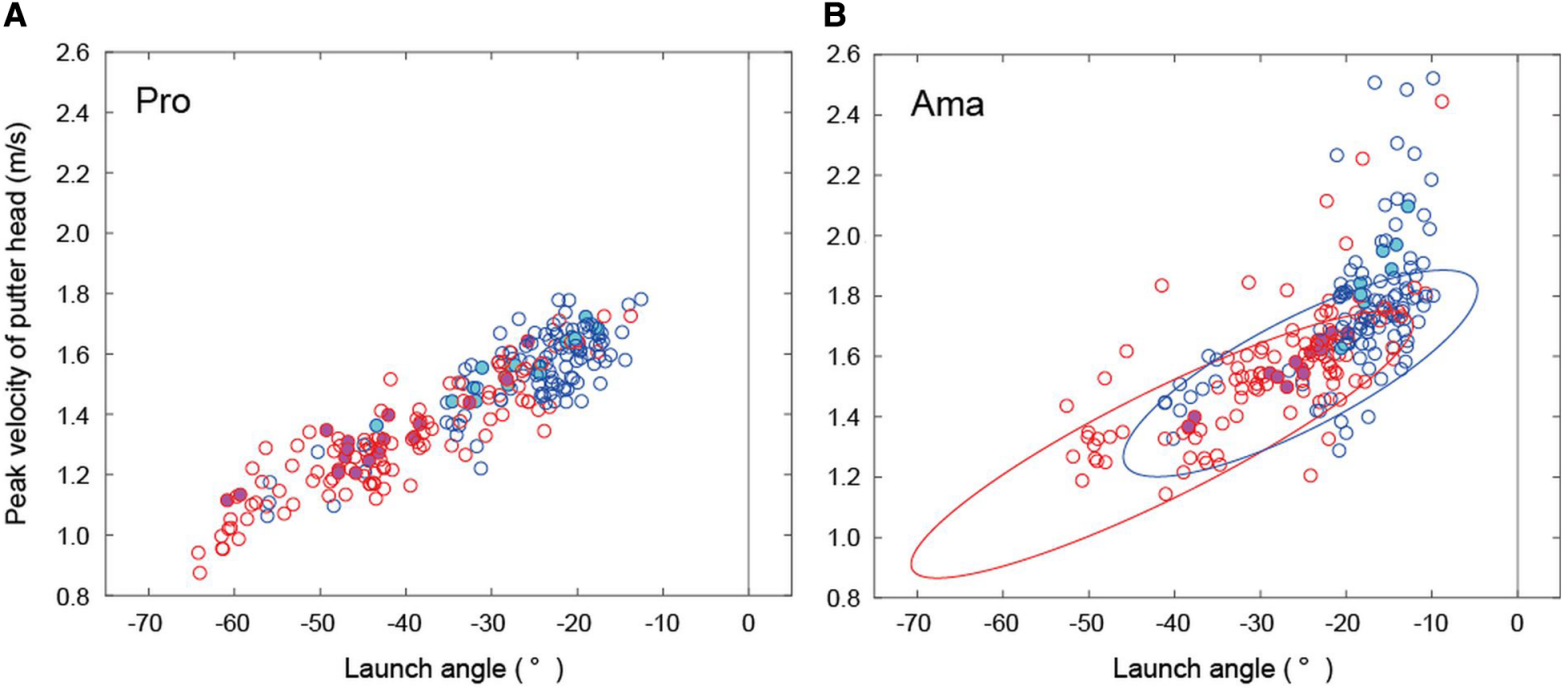
Relationship between ball launch angle and putter head peak velocity in both groups. Blue indicates trials without feedback, red indicates trials with feedback, cyan-filled markers indicate hole-in trials without feedback, and magenta-filled markers indicate hole-in trials with feedback. The two ellipses in (**B**) are overlaid with the probability ellipses (95%) of the professionals’ results. Negative values indicate the left side of the ball-hole line and positive values indicate the right side.

Figure 4 presents ball launch angles for all trials. The average and standard deviation values are listed in Supplementary Table S2. The ANOVA results indicated that the interaction was significant (*F*_1,22_ = 3.20, *p* = 0.09, *f* = 0.38). Simple-effects testing indicated that the launch angle in the FB condition was larger than that in the no FB condition for both groups (pro: *F*_1,22_ = 47.64, *p* = 6.24 × 10^−7^, *f* = 1.47, ama: *F*_1,22_ = 19.13, *p* = 2.42 × 10^−4^, *f* = 0.93). Additionally, both the no FB (*F*_1,22_ = 5.28, *p* = 0.03, *f* = 0.49) and FB conditions (*F*_1,22_ = 12.56, *p* = 0.002, *f* = 0.76) were significant for both groups; the aim angles of professionals and amateurs significantly differed, and the aim angles of professionals were larger than those of amateurs. The main effects are described in the supplementary text.

Figure 4 presents peak velocities for all trials. The average and standard deviation values are listed in Supplementary Table S2. The ANOVA results indicated that the interaction was not significant. However, the main effects of group (*F*_1,22_ = 6.31, *p* = 0.02, *f* = 0.54) and condition (*F*_1,22_ = 16.67, *p* = 4.93 × 10^−4^, *f* = 0.87) were significant; the peak velocity of professionals was lower than that of amateurs, regardless of condition, and the peak velocity in the FB condition was lower than that in the no FB condition, regardless of skill level.

Table 2 presents the results of the ball-launch angle and putter head peak velocity for the hole-in trials. Regarding the ball launch angle in the hole-in trials, Welch's t-test revealed significant differences between professionals and amateurs in both conditions (no FB: *t*_23_ = 5.60, *p* = 1.07 × 10^−5^, *d* = 0.76, with FB: *t*_29_ = 5.79, *p* = 2.86 × 10^−6^, *d* = 0.77). Regarding the peak velocity in the hole-in trials, Welch's *t*-test revealed that there were significant differences between professionals and amateurs in both conditions (no FB: *t*_23_ = 5.73, *p* = 0.0001, *d* = 0.77, with FB: *t*_29_ = 5.46, *p* = 6.98 × 10^−6^, *d* = 0.71).

**Table 2:** The ball launch angle and the peak velocity of the putter head in hole-in trials.

	Ball launch angle (°)				Peak velocity (m/s)			
	No feedback		With feedback		No feedback		With feedback	
	Pro	Ama	Pro	Ama	Pro	Ama	Pro	Ama
Average	−26.87	−16.56	−42.52	−26.13	1.55	1.87	1.33	1.58
SD	6.56	2.63	10.32	5.82	0.09	0.14	0.15	0.11
*n*	17	8	19	14	17	8	19	14

These results present the statistics of the hole-in-trials, as shown in Figure 4.

## 4. Discussion

To clarify why the aim of golf putting in risky situations differ according to skill level, a steep-slope golf putting task was used and tour professionals and intermediates were asked to play with no visual feedback of outcomes (no FB), followed by playing with FB. The aim of the task was to make the ball enter the hole in one shot. As a task constraint, the participants were told that if the ball did not enter the hole, they should try to get it as close to the hole as possible. The results of this study suggest that amateurs' failure to find low-risk routes lies in their decision-making.

This study confirmed that ball trajectories differed significantly based on skill level (Figures 1A–D). That is, the highest point in the mediolateral direction (MLD) of the professionals' ball trajectory was higher than that of the amateurs, particularly in the FB condition (Figure 2A). Additionally, the professionals' ball speed near the hole was approximately half that of amateurs (Figure 2B). Due to the limitations of the experimental set-up, even if the ball speed was close to zero near the hole, the balls that did not enter the hole did not stop on the platform either. However, in principle, the ball speed being close to zero near the hole indicated that less risky angles and velocities were chosen.

The results from the combination of ball launch angles and peak putter head speed in hole-in trials that generated ball trajectories indicated redundancy in the solution (Table 2). Additionally, the hole-in trials indicated that professionals chose a combination of speed and angle that would lower the ball speed near the hole when they did not make a hole-in. When all angles and velocities were compared between the groups, the professionals had a larger launch angle and a lower peak velocity under both conditions than the amateurs (Figures 3A, 4). This suggests that amateurs' decision-making differed from that of professionals.

However, because kinematics such as angle and velocity include motor variability (21, 22), it cannot be said that amateurs' decision-making is worse than that of professionals by only measuring their motor execution. Therefore, this study measured participants' aim before hitting. Analysis of the aim angles indicated that the aim angles differed between professionals and amateurs under both conditions. The difference in professionals' and amateurs' aims under the no FB may emanate from the amateurs' inaccurate perception of the slope (6), and/or failure to select the low-risk routes.

Participants could see the roll of the ball they hit during each trial with FB. Therefore, even if the amateurs did not perceive the slope properly, they may change their aim in the FB condition to ensure that the hit ball stays close to the hole. However, it is noteworthy that the amateurs' aim angles were significantly smaller than that of the professionals, even with FB. Because the launch angle of amateurs was smaller than that of professionals in the no FB condition, for amateurs to achieve the task constraints in the FB condition, it was necessary to change to a higher launch angle than professionals based on the lack of FB. Nevertheless, the differences in the values of the aim angles tended to be greater for professionals than amateurs. Therefore, it became clear that the professionals adjusted and fine-tuned their aim more in search of a route that was more suitable for the task (Figure 3B). This result also confirmed that amateurs’ ability to respond flexibly to tasks was lower than that of professionals.

Amateurs did not perform well in the tasks, even with FB, for two possible reasons. First, amateurs may not be able to select the route chosen by professionals. Professionals have substantial experience in dealing with novel situations, such as this study's task, and intermediate-level amateurs likely have little experience in this regard. Although there were constraints on the task, the participants were free to set up their aims. A study of motor learning in novices reported that intra-individual biases prevented learners from approaching optimal solutions during motor task learning (1). Therefore, amateurs may not recognize the multiple ways to achieve their goals. Second, for amateurs, hitting at a smaller launch angle and higher velocity may be optimal. To launch the ball in higher directions on steep slopes, it is necessary to precisely control the speed of the putter head. Although this study did not investigate motor variability, previous studies reported that non-experts' variation in speed control was greater than that of experts and that it is considered difficult for amateurs to finely adjust speed [e.g., (17, 23)]. It has also been suggested that variabilities in motor performance should be considered during motor planning (24, 25). For professionals, a larger aiming angle and slower speed reduce risks; however, for unskilled amateurs, aiming at a smaller angle may be less risky. This issue requires further examination in future research.

A previous study suggested that amateurs do not play well on steep slopes because they underestimate the slope (6). In addition, this study concluded that amateurs' failure to objectively select low-risk routes on steep slopes is also due to their decision-making. This study's findings suggest to golf coaches and athletes that there are many different routes in steep putting, as observed in the professional performance in this study. If the golfer hits the ball in a higher direction on steep slopes, precise club-head speed control is required. When golfers practice putting on steep inclines, it is important to attempt several different routes (different launch directions and ball speeds).

## Data Availability

The original contributions presented in the study are included in the article/**Supplementary Materials**, further inquiries can be directed to the corresponding author/s.

## References

[1] Ota K Shinya M Kudo K. Sub-optimality is motor planning is retained throughout 9 days practice of 2250. Sci Rep. (2016) 6:37181. 10.1038/srep37181 27869198 PMC5116677

[2] Pezzulo G Barca L Bocconi AL Borghibe AM. When affordances climb into your mind: advantages of motor simulation in a memory task performed by novice and expert rock climbers. Brain Cogn. (2010) 73:68–73. 10.1016/j.bandc.2010.03.002 20381226

[3] Seifert L Wattebled L L'Hermette M Bideault G Herault R Davids K. Skill transfer, affordance and dexterity in different climbing environments. Hum Mov Sci. (2013) 32:1339–52. 10.1016/j.humov.2013.06.006 24055363

[4] Boschker MSJ Bakker FC Michaels CF. Memory for the functional characteristics of climbing walls: perceiving affordances. J Mot Behav. (2002) 34:25–36. 10.1080/00222890209601928 11880247

[5] Ziv G Lindor R. Gaze behavior in golf putting—a review. Int J Golf Sci. (2019) 7. https://www.golfsciencejournal.org/article/10140-gaze-behavior-in-golf-putting-a-review

[6] Hasegawa Y Okada A Fujii K. Skill differences in a discrete motor task emerging from the environmental perception phase. Front Psychol. (2020) 12:Article 697914. 10.3389/fpsyg.2021.697914 PMC8517186 34659013

[7] Dias G Couceiro MS Barreiros J Clemente FM Mendes R Martins FML. Distance and slope constraints: adaptation and variability in golf putting. Motor Control. (2014) 18:221–43. 10.1123/mc.2013-0055 24280087

[8] Craig CM Delay D Grealy MA Lee DN. Guiding the swing in golf putting. Nature. (2000) 405(6784):295–6. 10.1038/35012690 10830947

[9] Delay D Nougier V Orliaguet J Coello Y. Movement control in golf putting. Hum Mov Sci. (1997) 16:597–619. 10.1016/S0167-9457(97)00008-0

[10] Mathers JF Grealy MA. Motor control strategies and the effects of fatigue on golf putting performance. Front Psychol. (2014) 4:Article1005. 10.3389/fpsyg.2013.01005 24454298 PMC3888943

[11] Karlsen J Smith G Nilsson J. The stroke has only a minor influence on direction consistency in golf putting among elite players. J Sports Sci. (2008) 26:243–50. 10.1080/02640410701530902 17917952

[12] Hume PA Keogh J Reid D. The role of biomechanics in maximising distance and accuracy of golf shots. Sports Med. (2005) 35:429–49. 10.2165/00007256-200535050-00005 15896091

[13] Schimdt RA Lee TD Winstein CJ Wulf G Zelaznick HN. Motor control and learning: A behavioral emphasis. Champaign, IL: Human Kinetics (2019).

[14] Van Lier W Van Der Kamp J Savelsbergh GJP. Gaze in golf putting: effects of slope. Int J Sport Psychol. (2010) 41(2):160–76.

[15] Jackson KM. Fitting of mathematical functions to biomechanical data. IEEE Trans Biomed Eng. (1979) 26:122–4. 10.1109/TBME.1979.326551 761932

[16] Winter DA. Biomechanics and motor control of human movement, (2nd ed.). New York: Wiley (1990).

[17] Hasegawa Y Fujii K Miura A Yamamoto Y. Resolution of low-velocity control in golf putting differentiates professionals from amateurs. J Sports Sci. (2017) 35:1239–46. 10.1080/02640414.2016.1218037 27686139

[18] Hasegawa Y Fujii K Miura A Yokoyama K Yamamoto Y. Motor control of practice and actual strokes by professional and amateur golfers differ but feature a distance dependent control strategy. Eur J Sport Sci. (2019) 19:1204–13. 10.1080/17461391.2019.1595159 30922210

[19] Faul F Erdfelder E Lang AG Buchner A. G*power 3: a flexible statistical power analysis program for the social, behavioral, and biomedical sciences. Behav Res Methods. (2007) 39:175–91. 10.3758/bf03193146 17695343

[20] Cohen J. Statistical power analysis for the behavioral sciences, (2nd ed.). Hillsdale, NJ: Lawrence Erlbaum Associates (1988).

[21] Harris CM Wolpert DM. Signal-dependent noise determines motor planning. Nature. (1998) 394:780–4. 10.1038/29528 9723616

[22] van Beers RJ Haggard P Wolpert DM. The role of execution noise in movement variability. J Neurophysiol. (2004) 91:1050–63. 10.1152/jn.00652.2003 14561687

[23] Tanaka H Iwami M. Estimating putting outcomes in golf: experts have a better sense of distance. Percept Mot Skills. (2018) 125:313–28. 10.1177/0031512518754467 29357738

[24] Kudo K Tsutui S Ishikura T Ito T Yamamoto Y. Compensatory coordination of release parameters in a throwing task. J Mot Behav. (2000) 32:337–45. 10.1080/00222890009601384 11114227

[25] Takiyama K Hirashima M Nozaki D. Prospective errors determine motor learning. Nat Commun. (2014) 6:5925. 10.1038/ncomms6925 PMC4316743 25635628

