# Supplementary material for: Can golfers choose low-risk routes in steep putting based on visual feedback of ball trajectory?
Source: Front Sports Act Living. 2023 Aug 22;5:1131390. doi: 10.3389/fspor.2023.1131390 (PMC10477702; doi:10.3389/fspor.2023.1131390)
Supplement: Supplementary file 2 [file Datasheet2.docx]

Supplementary Material

# Supplementary Text

# Full results

## Ball trajectory and ball speed near the hole

Figure 1A-D shows the ball trajectories for all. The ANOVA results for the highest point for APD (Figure 1E) showed that the interaction was not significant. However, the main effect of condition (F_1,22_ = 53.00, p = 0.002, f = 0.74, 1-β = 0.99) was significant; the highest point for the APD with FB condition was larger than that of the no FB condition, regardless of skill level. The main effect of the group was not significant.

The ANOVA results for the highest point for MLD (Figure 1E) showed that the interaction was significant (F_1,22_ = 5.67, p = 0.03, f = 0.51, 1-β = 0.99). Simple-effects testing indicated that the highest point for MLD of professionals with the FB condition was higher than that of amateurs (F_1,22_ = 11.09, p = 0.003, f = 0.71, 1-β = 0.97). The highest point for MLD of professionals and amateurs was higher with FB than with no FB (pro: F_1,22_ = 38.66, p = 2.91×10^-6^, f = 1.33, 1-β = 1.00, ama: F_1,22_ = 8.13, p = 0.009, f = 0.61, 1-β = 0.99).

The ANOVA results for ball speed near the hole (Figure 1F) showed that the interaction was not significant. However, the main effects of group (F_1,22_ = 12.97, p = 0.002, f = 0.76, 1-β = 0.98) and condition (F_1,22_ = 62.81, p = 6.90×10^-8^, f = 1.68, 1-β = 1.00) were significant, the ball speed of professionals was lower than that of amateurs, and the ball speed in the FB condition was lower than that in the no FB condition, regardless of skill level.

## Aim angle and the difference values between trials

The results of the ANOVA for the aim angle (Figure 2A) showed that the interaction tended to be significant (F_1,22_ = 3.25, p = 0.09, f = 0.38, 1-β = 0.94). Simple-effects testing indicated that the aim angle in the FB condition was larger than that in the no FB condition in both groups (pro: F_1,22_ = 41.87, p = 1.64×10^-6^, f = 1.38, 1-β = 1.00, ama: F_1,22_ = 15.38, p = 7.30×10^-4^, f = 0.84, 1-β = 1.00). In addition, both the no FB (F_1,22_ = 7.14, p = 0.01, f = 0.57, 1-β = 0.99) and FB conditions (F_1,22_ = 15.41, p = 7.23×10^-4^, f = 0.84, 1-β = 1.00) in both groups were significant; the aim angles of professionals and amateurs were significantly different, and the aim angles of professionals were larger than those of amateurs. In addition, the main effects of group (F_1,22_ = 14.25, p = 0.001, f = 0.80, 1-β = 0.99) and condition (F_1,22_ = 53.00, p = 2.72×10^-7^, f = 1.57, 1-β = 1.00) were significant.

The ANOVA results for the difference values between trials of the aim angle (Figure 2B) showed that the interaction was not significant. However, the main effect of the group tended to be significant (F_1,22_ = 3.02, p = 0.09, f = 0.37, 1-β = 0.93); the difference in values for professionals was larger than that for amateurs. Additionally, the main effect of the variation was significant (F_1,22_ = 7.74, p = 0.01, f = 0.59, 1-β = 0.99). The results of multiple comparisons are shown in Supplementary Table 1.

## Ball launch angle, the peak speed of the putter head, and hole-in trials

Figure 3 shows all trials of the ball launch angles. The average and standard deviation values are listed in Supplementary Table 2. The ANOVA results showed that the interaction was significant (F_1,22_ = 3.20, p = 0.09, f = 0.38, 1-β = 0.94). Simple-effects testing indicated that the launch angle in the FB condition was larger than that in the no FB condition for both groups (pro: F_1,22_ = 47.64, p = 6.24×10^-7^, f = 1.47, 1-β = 1.00, ama: F_1,22_ = 19.13, p = 2.42×10^-4^, f = 0.93, 1-β = 1.00). In addition, both the no FB (F_1,22_ = 5.28, p = 0.03, f = 0.49, 1-β = 0.99) and FB conditions (F_1,22_ = 12.56, p = 0.002, f = 0.76, 1-β = 1.00) in both groups were significant; the aim angles of professionals and amateurs were significantly different, and the aim angles of professionals were larger than those of amateurs. In addition, the main effects of group (F_1,22_ = 11.56, p = 0.003, f = 0.72, 1-β = 0.97) and condition (F_1,22_ = 63.57, p = 6.24×10^-8^, f = 1.70, 1-β = 1.00) were significant.

Figure 3 also shows all trials of the peak velocity. The average and standard deviation values are listed in Supplementary Table 2. The ANOVA results showed that the interaction was not significant. However, the main effects of group (F_1,22_ = 6.31, p = 0.02, f = 0.54, 1-β = 0.83) and condition (F_1,22_ = 16.67, p = 4.93×10^-4^, f = 0.87, 1-β = 1.00) were significant; the peak velocity of professionals was lower than that of amateurs, regardless of condition, and the peak velocity in the FB condition was lower than that in the no FB condition, regardless of skill level.

Table 1 shows the results of the ball-launch angle and putter head peak velocity for the hole-in trials. Regarding the ball launch angle in the hole-in trials, Welch’s t-test revealed significant differences between professionals and amateurs in both conditions (no FB: t_23_ = 5.60, p = 1.07×10^-5^, d = 0.76, with FB: t_29_ = 5.79, p = 2.86×10^-6^, d = 0.77). As for the peak velocity in the hole-in trials, Welch’s t-test revealed that there were significant differences between professionals and amateurs in both conditions (no FB: t_23_ = 5.73, p = 0.0001, d = 0.77, with FB: t_29_ = 5.46, p = 6.98×10^-6^, d = 0.71).

# Supplementary figure

#
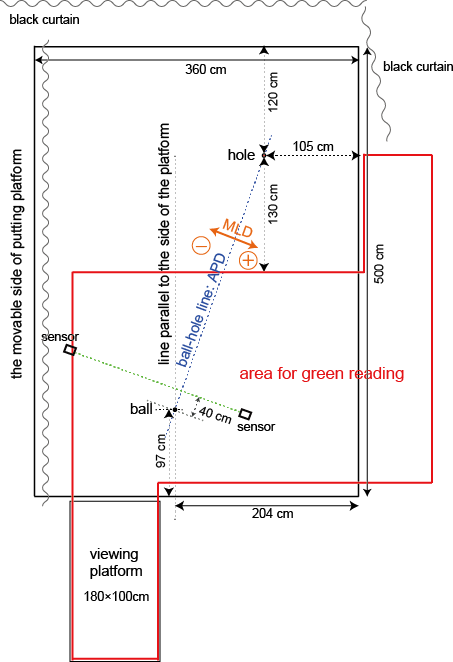


**Supplementary Figure 1. Experiment setting.** It represents the pattern diagram of the experiment setting. The experiment area where the putting platform was set was surrounded by curtains, and care was taken not to give the participants reference frames. The area surrounded by the red line indicates where participants could move freely to read the green. Two sensors were placed so that the shutter goggles would be activated when the ball passed the line 40 cm in front of where it was set. APD, anteroposterior direction; MLD, mediolateral direction.

# Supplementary Tables

**Supplementary Table 1. The result of multiple comparisons for the difference values between trials of the aim angle.**


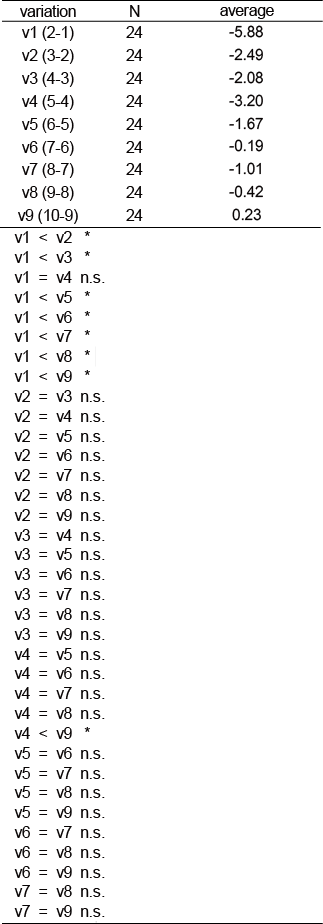


Note. MSe = 10.99, * p<.05

**Supplementary Table 2. The average ball launch angle and the average peak velocity of the putter head.**


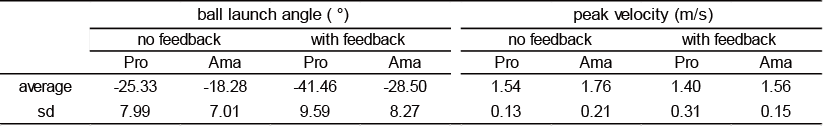


Note. Sd = standard deviation.
